# Effects of Heat Input and Intertrack Overlap on the Microstructure and Properties of Inconel 686 Weld Overlays

**DOI:** 10.3390/ma17133315

**Published:** 2024-07-04

**Authors:** Seyedmohammad Tabaie, Zahra Khodamoradi, Trevor Greene, Michael J. Benoit

**Affiliations:** 1 School of Engineering, University of British Columbia, Kelowna, BC V1V 1V7, Canada; zahra.khodamoradi@ubc.ca (Z.K.); michael.benoit@uwaterloo.ca (M.J.B.); 2 Teck Resources Limited, Trail, BC V1R 4L8, Canada; trevor.greene@teck.com; 3 Centre for Advanced Materials Joining, Department of Mechanical & Mechatronics Engineering, University of Waterloo, Waterloo, ON N2L 3G1, Canada

**Keywords:** cladding, weld overlay, Inconel 686, process optimization, dilution, microsegregation

## Abstract

The objective of this study was to investigate how weld overlays with nickel superalloys are important for the integrity, due the high temperatures and corrosive environments that can be experienced in mineral processing environments, of mining and processing equipment. The Ni-Cr-Mo superalloy Inconel 686 overlays are fabricated through automatic gas metal arc welding with variations in arc voltage and travel speed (i.e., heat input), and they have overlap between adjacent weld tracks for applications in the mining and minerals sector. The impact of variations in the process parameters and the size of the weld overlapping on the dilution, solidification morphology, microsegregation, and microhardness were investigated. Both geometric and chemical composition definitions were used to quantify the extent of the weld dilution. Subsequently, the weld geometry and dilution were correlated with the solidification microstructure and phase transformations. The maximum dilutions were measured to be 13.63% (1/2 overlap, 5.96 kJ·cm^−1^) and 15.39% (1/3 overlap, 4.77 kJ·cm^−1^), which shows that less of an overlap increases the dilution level. Scanning electron microscopy and chemical composition analysis revealed that an increase in weld heat input and dilution level led to higher levels of microsegregation for Mo and Cr, as well as the volume fraction of Mo- and Cr-rich phases in the interdendritic/intercellular regions in the overlay layer. Analysis of the weld overlays in the current study revealed strong and unprecedented connections between the weld overlay process conditions, the resultant metallurgy (i.e., dendrite arm spacing, microsegregation, and phase formation), and the hardness of the overlay. It was concluded that the optimal weld overlays in the processing window studied in this investigation were fabricated at mid-level heat inputs (i.e., 4–5 kJ·cm^−1^) and a 1/2 track overlap.

## 1. Introduction

Smelting furnace off-gasses can contain many corrosive species, including sulfides, sulfates, acids, and metallic-chloride vapors. The waste heat boilers used to cool these gasses must be protected to maintain the integrity of the boiler. Savings on material and production costs can be achieved using weld overlay techniques during the production of critical components for the mining industry [1]. Weld overlays, also referred to as cladding or hardfacing is a surface treatment procedure employed to apply one or more layers of a corrosion- or wear-resistant material onto a base material, typically a more economical substrate material such as steel. Thus, using weld overlays to apply high-performance materials such as nickel superalloys can enhance the mechanical characteristics and corrosion resistance of boiler components. Several arc welding techniques are widely used as heat sources for weld overlay operations in mineral processing applications, including gas tungsten arc welding (GTAW), gas metal arc welding (GMAW), and shielded metal arc welding (SMAW) [2,3,4].

Ni–Cr–Mo alloys, such as Inconel 686 (IN686), have been used as weld overlay filler metal on the internal surface of metallic waste heat boilers (e.g., KIVCET), which are commonly used for the smelting and reduction of zinc and lead concentrates. In addition, they operate at high temperatures because of their outstanding blend of mechanical characteristics, elevated-temperature corrosion/oxidation resistance, and ease of welding [3,5]. Thornton and Cooper [6] reported that the substantial level of alloying elements in the IN686 superalloy provides a high level of general corrosion resistance. Moreover, the combination of chromium (Cr) and molybdenum (Mo) improves resistance to both pitting and crevice corrosion, while the addition of tungsten (W) has also been shown to improve the resistance of IN686 to the localized corrosion in weld overlays on duplex stainless steels. Adamiec [7] evaluated the effects of composition on the corrosion properties of IN686 and IN625 overlays, particularly the amounts of Cr and Mo, which provide good resistance to oxidization and reduce environmental damage. It was revealed that the Cr formed a surface oxide layer, while the Mo increased resistance to localized corrosion by promoting the re-passivation of the oxide film. Arulmurugan and Manikandan [8] welded IN686 alloy by the GTAW technique and found the restricted amount of carbon (C) in this superalloy (i.e., ≤0.01 wt.%) amplified the corrosion resistance through the inhibition of grain boundary precipitation in the heat-affected zone (HAZ) of welded regions. Silva et al. [9] studied the weld overlay of IN686 by the GTAW technique and reported that brittle topologically close-packed (TCP) phases (e.g., σ, P, and μ phases) were formed due to the segregation of Mo, W, and Cr during solidification, in which their amounts exceed the solubility limit of the Ni matrix. Similar results were also reported by Volpi and Serra [10], noting that it is desirable to avoid the formation of TCP phases during solidification as these phases are known to adversely affect corrosion properties. Mina et al. [11] also fabricated IN686 weld overlays using GTAW, as well as by changing the wire feed speed (WFS) and the welding pattern, but they considered welding at a constant heat input (HI). They also reported that using GTAW on the IN686 overlay resulted in high amounts of dilution, ranging from 14.3% to 33.3% as the WFS was lowered from 8.5 to 4.5 m·min^−1^. Consequently, increasing dilution increased the amount of iron (Fe) in the primary Ni solid phase during solidification, the solubility of W, Cr, and Mo slightly decreased in a solid solution, meaning their partition coefficients (k) also decreased. 

Singh Singhal and Kumar Jain [12] studied the cladding of ferrous and non-ferrous metals by the GMAW method and reported that cladding by the GMAW enhances the weld overlay corrosion resistance by reducing the amount of dilution and increasing the surface properties when compared to GTAW. Najafi et al. [13] reported that the GMAW method for the IN625 overlay induces a maximum of a 13% compositional dilution (which was determined by measuring the Fe content in the weld overlay), and this was less than the minimum dilution observed for GTAW overlays (more than 20% on average). Tabaie et al. [14] investigated the effect of the GMAW parameters on the clad layer of IN686 superalloys, concluding that the defect-free clad samples with optimum surface quality resulted from the mid-level weld heat input (4–5 kJ·cm^−1^) and a WFS of 8 m·min^−1^. In addition, they found that low levels of welding dilution could reduce the microsegregation of Cr and Mo and the formation of TCP particles. Volpi and Serra [10] reported that the dilution amount can vary by altering the preheat and interpass temperatures, as well as the arc oscillation during welding. Xu et al. [15] studied the effects of track overlap on the surface quality of laser cladding layers of the IN718 superalloy and found that lower track overlap amounts produced clad layers with no cracks or gaps, as well as that the grains were refined at the overlapping areas.

While there has been substantial research on weld overlays, noticeable gaps exist in the literature concerning the interaction between track overlap and automatic GMAW process parameters, specifically regarding their influence on the solidification behavior in the automatic GMAW overlays of the IN686 superalloy. The analysis of weld overlays in the current study aims to reveal unprecedented connections between the weld overlay process conditions, the resultant metallurgy (i.e., dendrite arm spacing, microsegregation, and phase formation), and the hardness of the overlay. This study aims to address this gap by evaluating the effects of weld overlay track overlap and variations in GMAW parameters on the quality and microstructure of IN686 overlays on an ASME SA516-Gr.70 plate.

## 2. Materials and Methods

### 2.1. Materials

Commercially available Inconel 686 wire (AWS ER-NiCrMo-14) supplied by Special Metals Co. (Hartford, NY, USA). with a diameter of 1.143 mm was used as the filler metal in this study. The base materials utilized for this purpose consisted of plates made from low-carbon steel conforming to the ASME SA516-Gr.70 specifications, with dimensions of 152.4 × 152.4 × 6.35 mm^3^. The welding surface of each plate was ground prior to applying the overlay layer. The chemical composition of the as-received materials, as measured from multiple energy dispersive X-ray spectroscopy (EDS) analyses in large areas of analysis, is shown in Table 1.

### 2.2. Design of the Experiment

**Figure 1:**
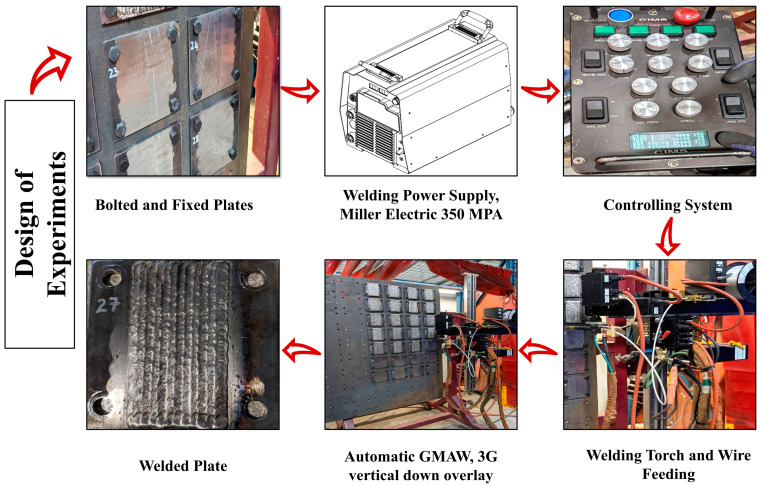
A schematic diagram of the weld overlay experimental setup.

Figure 1 depicts the experimental arrangement and the sequential steps involved in the weld overlay procedure. A design of experiment (DoE) methodology was employed for the weld overlay process utilizing Taguchi’s L9 (3^2^) orthogonal array (extracted from a L27 design model). In this approach, the arc voltage (V) and torch travel speed (TS) were changed, each at three different levels, resulting in a total of 9 distinct overlay conditions, as detailed in Table 2. In addition, the overlap between adjacent weld tracks in the overlay was also varied at two levels (i.e., 1/2 and 1/3 of the previous bead width, herein designated as overlap half or “OH” and overlap third or “OT”, respectively). Therefore, 18 sample conditions or two groups of Taguchi’s L9 (32) orthogonal array were welded in this investigation. The overlays were manufactured using a Miller Electric 350 MPA power supply, which was equipped with an automated wire feeding system, with a constant WFS of 8 m·min^−1^ and a welding current (I) of 190 A. The power supply was integrated with an Encompass Machines Inc. semi-automated MIG overlay system (MOS) to regulate and control the movement of the torch during the overlay process. The weld overlays were fabricated in the 3G vertical down position (as a practical position used for automatic weld overlay in boilers) using argon as a shielding gas with a flow rate of 18 L·min^−1^. As shown in Figure 1, a vertical wall was provided to fix and bolt the plates during welding. To minimize defects during the welding process, a triangular weave technique was employed, featuring a horizontal arc amplitude of 3 mm. The overlay procedure did not involve pre-heating the base plate, although an interpass temperature of 200–300 °C was measured between the deposition of the adjacent tracks. The weld overlays had an overall width of approx. 100 mm, which was composed of 12 (overlap half) or 10 (overlap third) weld tracks that were deposited along the full length of the plates. The welding was conducted in the welding shop at ambient temperature. Further details of the welding procedure can be found in [14].

Table 2 provides all the weld overlay conditions that were studied. Weld overlay plates were coded based on the overlap (OH or OT) and the heat input (HI) per unit length, which was calculated by Equation (1):

(1) $$HI=\frac{60\times I\times V\times \eta }{1000\times TS}\;\left(\frac{kJ}{cm}\right).$$

In which η is the process arc efficiency, which was assumed to be ~0.85 for GMAW [16].

### 2.3. Microstructure Analysis

Conventional metallographic preparation and analysis was performed on weld overlay cross-sections that were cut from plates transverse to the weld direction. Two large samples containing at least three weld tracks (beads) were considered. A Keyence digital microscope was used to evaluate the weld macrostructure. Nital (2%) and Marble reagent were employed to expose the base metal (BM) section, while the microstructure of the IN686 weld metal (WM) section was assessed through electrolytic etching using a 10% solution of chromic acid at 2 volts for a duration of 15 to 20 s.

**Figure 2:**
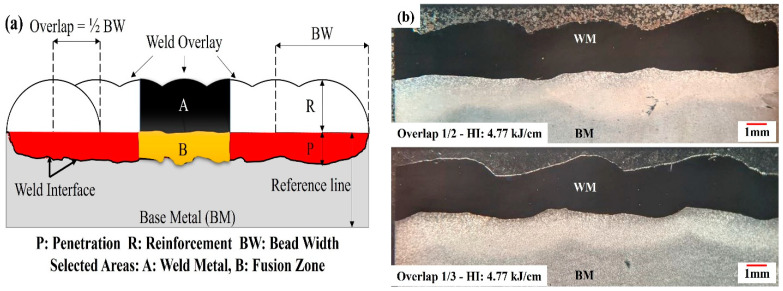
(**a**) A schematic of the weld geometry characteristics and the selected areas for geometric dilution calculation, and (**b**) example macrographs of each weld overlap condition.

Macrographs from the overlay cross-sections were analyzed using ImageJ 2.9 software, and the weld geometrical features were measured. As shown in Figure 2, the reference line corresponds to the original top surface of the substrate plate (Figure 2a), and an example macrograph for each weld overlap is shown in Figure 2b. According to the reference line, the weld reinforcement (R), bead width (BW), and penetration (P) were measured. Furthermore, the geometric dilution (d_g_) of each weld overlay plate was calculated by Equation (2), with the areas shown in Figure 2:

(2) $$d_{g}\;(\%)=\frac{B}{A+B}.$$

In which B is the fusion zone area beneath the reference line and A is the area of the weld metal above the reference line. The concentrations of Fe in the filler metal (the nominal Fe content in the wire, Fe_fm_), the measured Fe in the weld metal (close approximation to the welded/fusion zone in region B, Fe_wm_), and in the unaffected BM (Fe_BM_), were obtained by energy dispersive spectroscopy (EDS) spot measurements. The compositional dilution (d_c_) was calculated for select samples with varying levels of d_g_ using Equation (3):

(3) $$d_{c}\;(\%)=\frac{{Fe}_{wm}-{Fe}_{fm}}{{Fe}_{BM}-{Fe}_{fm}}.$$

A Tescan Mira 3 XMU field emission scanning electron microscope (FE-SEM), Brno, Czech Republic, equipped with an EDS detector was used for the microstructure evaluation of the base metal and the weld metal. The dendrite arm spacing (DAS) was measured from both SEM and high-magnification optical micrographs for several conditions to calculate the cooling rate. Moreover, each overlay’s fusion zone chemical composition was analyzed by EDS analysis (line scan and spot analysis) over the large areas where the filler metal melted and diffused into the BM, and this was conducted because it is the location where most of the mixing/dilution occurs; therefore, we expected to see differences in the solidification structure and phase evolution for different welding conditions. During SEM analysis, both backscattered electron (BSE) and secondary electron (SE) detectors were employed to detect and analyze the secondary phases. ImageJ software was used to measure the volume fraction of particles formed in the weld microstructure. ASTM E1245 standard [17] requirements and recommendations were followed for sampling and measuring secondary precipitates. Therefore, all the results were analyzed according to the ASTM E1245 standard at the 95% confidence level.

### 2.4. Thermo-Calc Analysis

The commercially available calculation of phase diagram (CALPHAD) software package Thermo-Calc (version 2022b) with the TCNI11 Nickel-based alloys database was used to calculate and simulate the non-equilibrium solidification and phase evolution for different dilution levels. The chemical composition within the fusion zone (i.e., Region B in Figure 2), as analyzed by EDS point measurements, was used as an input for the simulations and compared with the literature. Non-equilibrium solidification was modeled using the Scheil equation up to a liquid fraction of 0.0001%.

### 2.5. Microhardness Measurement

The hardness of various microstructure zones within the cross-section of the overlay (i.e., the weld metal (WM), fusion zone (FZ), heat-affected zones (HAZs), and base metal (BM)) was analyzed by Vickers micro indentations for all the different weld overlay sample conditions. A Wilson VH3100 automated Vickers hardness machine, Buehler (Glenview, IL, USA), was utilized to apply a load of 1 kg for a dwell time of 10 s for each measurement. To assess the variability in the measured hardness values, three to five indentations were conducted in each specific zone.

## 3. Results and Discussion

### 3.1. Welding Geometries and Dilution

Table 3 provides the measured dimensions of the weld beads across six chosen welded samples, as well as the maximum heat-affected zone (HAZ) thickness. These samples were selected to represent the minimum, maximum, and average values of the dg and dc for each overlap condition. It is worth noting that, in line with expectations, both dg and dc exhibited an increase as the heat input (HI) increased for both levels of weld overlap. Moreover, no indications of cracks and no lack of fusion were detected through visual inspections of the overlay surface or in the polished cross section for all the samples studied. For instance, the macrograph samples from each overlap amount with a heat input of 4.77 kJ·cm^−1^ are shown in Figure 2b.

The overlap of the weld beads strongly influences the dimensions of the multi-pass overlay. The weld reinforcement (R) increased with increasing bead overlap for a constant energy input (HI). Conversely, the penetration into the BM was reduced with increasing overlapping as the arc was directed more at the previously deposited bead and less at the substrate; a similar finding was reported for the GTAW of 309L stainless steel [18]. Moreover, it can be seen from the table that both P and R generally increased when the HI increased for both overlaps. The OT-welded samples also resulted in a smaller BW than the OH-welded samples for the same HI. Welded plates with OT have more of a contact area with the substrate, which facilitates greater heat transfer and faster cooling, thus hindering the molten weld metal from spreading on the substrate surface and producing a smaller BW. In addition, half of the weld track with the OH was deposited on the previous hot weld track (i.e., the interpass temperature = ~200 °C), which caused slower cooling rates and a wider bead. 

**Figure 3:**
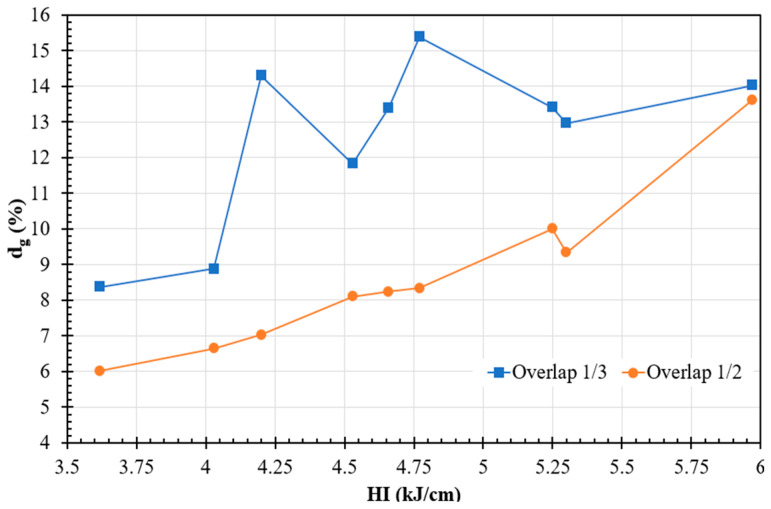
Geometric dilution (d_g_) variation as a function of weld heat input for two weld overlaps.

Figure 3 shows the variation in d_g_ against the HI for weld overlay plates with two different weld overlaps. The d_g_ exhibited a strong linear relationship with HI for the OH samples, which was influenced by the variation in V and TS. However, while there was a general positive trend between the dg and HI for OT, the relationship was not linear due to different weld geometries (i.e., R and P). For example, although OT4.77 had a higher d_g_ (15.39%) than OT5.97 (14.03%), OT5.97 produced a higher R (2.85 mm) than OT4.77 (2.4 mm), but both conditions had the same penetration level (0.6 mm). It should be noted that OT5.97 had a much lower travel speed (40.6 cm·min^−1^), allowing for more material deposition per unit length compared to OT4.77 (50.8 cm·min^−1^), which resulted in greater R. Since the current was roughly the same for both conditions, the penetration could be expected to be relatively constant. However, this behavior was not observed in the OH samples because the penetration increased with increasing heat input. 

Figure 3 also illustrates that the OH-welded plates exhibited lower dilution levels than the OT plates for all HI levels. This can be attributed to the fact that, spatially, the arc was shifted more over the previously deposited track with greater overlaps (i.e., OH); therefore, a larger proportion of the arc energy was employed to re-melt the previously laid weld bead rather than the steel substrate. Conversely, lower overlap values (i.e., OT) resulted in more arc energy being directed toward melting the steel substrate; hence, greater penetration and dilution.

Additionally, Table 3 provides the calculated dc values for six weld overlay plates, which were determined using Fe composition measurements that were obtained through EDS analysis (Equation (3)). When comparing d_g_ and d_c_, it became evident that there was generally a small difference in all the samples. Specifically, dc was found to be consistently lower than d_g_, with the exception of OT5.97, and this observation aligns with the findings reported by Silva et al. [9]. Consistent with the trend observed in d_g_, Table 3 emphasizes that the d_c_ was higher in the OT samples compared to the OH samples when the heat input (HI) remained constant, and the dilution generally increased with HI for a constant overlap amount.

**Figure 4:**
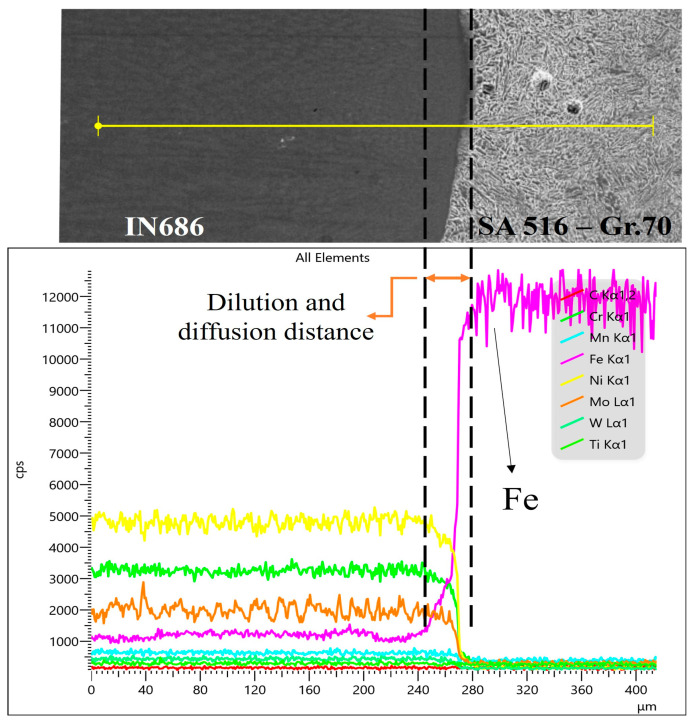
EDS line-scan analysis of the elemental variation from the SA 516-Gr.70 toward the IN686 alloy for (S#) OT4.77.

The chemical composition profile across the BM–weld interface, determined through EDS line scan, is shown in Figure 4 for the OT4.77 sample. The results revealed a transition in the Fe content (i.e., Fe dilution) in moving across the weld interface from the SA516-Gr.70 (BM) toward the IN686 weld metal (WM). Moreover, within the same range as the Fe transition, a reverse concentration gradient for Ni, Cr, and Mo was evident, and these changes occurred over a relatively short distance through the weld interface (~40 µm). The short diffusion distance in this region can be attributed to the rapid cooling that occurred. Mina et al. [11] reported that the alteration of local change in the chemical composition within the weld metal (even within a span of a few μm) induced by dilution could result in the formation of TCP phases, which are primarily composed of Fe, Mo, and Cr.

### 3.2. Microstructural Evolution

#### 3.2.1. The Base Metal—Heat-Affected Zone

**Figure 5:**
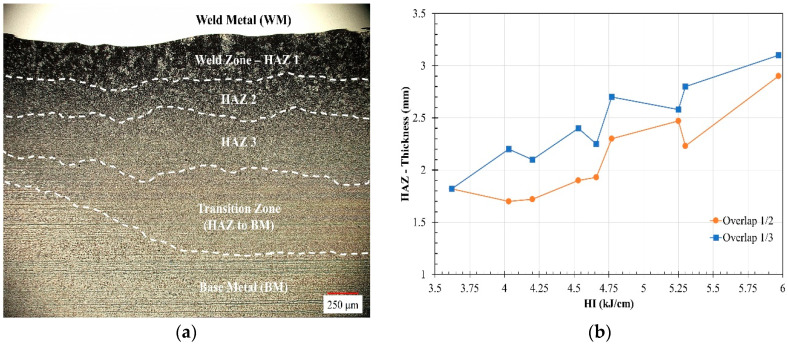
Optical micrographs of the OT4.77 sample in (**a**) all heat-affected zones and (**b**) the HAZ thickness variations within the welding heat input and weld overlap.

The weld heat input affected the base metal microstructure through the formation of different heat-affected zones (HAZs), as shown in Figure 5a for the OT4.77 sample. The HAZs of the SA516-Gr. 70 substrate after the GMAW overlay were previously analyzed entirely and reported by Tabaie et al. [14]. It was found that the HAZ is composed of three distinct microstructural regions, with the weld zone (WZ)-HAZ 1 and HAZ 2 exhibiting the presence of coarse grains, while refined grains were observed in HAZ 3 and the transitional zone.

The microstructure of the overlay substrate was predominantly influenced by the weld heat input, cooling rate, and the chemical composition alteration resulting from the Fe dilution into the weld metal. The variation in the maximum thickness of the entire HAZ, as measured by optical microscopy and as a function of the heat input (HI) and bead overlap, is shown in Figure 5b. The maximum HAZ thickness generally increased with increasing heat input for both weld overlap conditions. Furthermore, it was evident that the OT samples consistently had a larger HAZ compared to the OH samples, and this was due to the greater interaction area between the welding arc and substrate in the former. 

#### 3.2.2. The Weld Metal—Dendrite Arm Spacing and Cooling Rate

**Figure 6:**
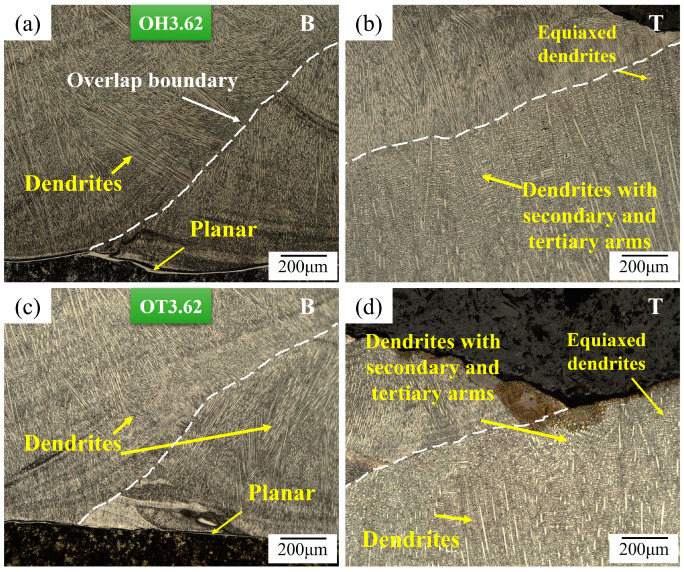
Optical micrographs of the overlays fabricated with the minimum heat input of 3.62 kJ·cm^−1^ for (**a**,**b**) OH and (**c**,**d**) OT samples at the (**a**,**c**) bottom surface (B) and (**b**,**d**) the top part (T) of the weld metal.

Figure 6 illustrates the solidification morphologies at two regions within the weld metal: near the weld interface (Bottom or B) and at the uppermost section of the weld metal (Top or T). While higher magnification images were utilized to measure the microsegregation and DAS, the lower magnification images presented in Figure 6 were employed to highlight the analysis areas. These observations (Figure 6a,b vs. Figure 6c,d) pertain to two different weld overlap conditions, where the minimum heat input (HI: 3.62 kJ·cm^−1^) was utilized. In the bottom regions of both weld overlaps, a mix of planar, cellular, and columnar dendritic grain formations can be observed, which are attributable to the rapid variation in the ratio of the thermal gradient (*G*) to solidification growth rate (*R*) (i.e., *G/R*) in this specific region. The top area contained an equiaxed dendritic structure with secondary and tertiary dendrite arms. This transition in grain morphology is attributed to a reduction in the thermal gradient in the weld metal during the solidification at the top of the overlay, in the overlapping with the next layer, and the thermal effect of a new weld track on the previous one (i.e., due to re-melting during the deposition of the subsequent weld track).

**Figure 7:**
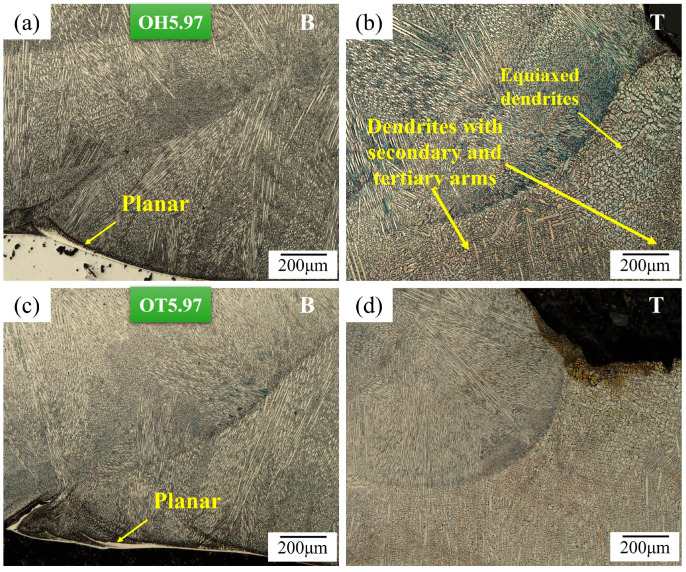
Optical micrographs of the weld metal in overlays that were welded using the highest heat input of 5.97 kJ·cm^−1^ for (**a**,**b**) OH and (**c**,**d**) OT samples at the (**a**,**c**) bottom surface (B) and (**b**,**d**) the top part (T) of the weld metal.

Figure 7 shows the solidification structures at the bottom and top regions of the weld metal in samples that were welded using the highest heat input (HI: 5.97 kJ·cm^−1^) for the two weld overlap conditions. The welded samples with the maximum weld heat input produced, due to generating more heat in the weld metal and reducing the thermal gradient (*G*), a larger area fraction of equiaxed dendrite near the top surface of the weld overlay (Figure 7b,d) when compared to the welded samples with the minimum weld heat input (Figure 6b,d).

In multiple-pass welding and overlapping, each new weld bead reheats and partially re-melts the previously deposited bead (in the overlapping area). The weld metal microstructure is highly dependent on the cooling rate of the overlay, which is, in turn, affected by the total heat input and overlap amount. The deposition on previously deposited weld bead that had not returned to ambient temperature resulted in a reduction in the cooling rate, and this effect was more pronounced for larger (i.e., OH) overlaps compared to single-pass welding or shorter overlapping values (i.e., OT samples); however, the OT samples had a greater contact area with the substrate, which led to faster cooling. 

Investigating the influence of heat input on the cooling rate requires a comprehensive analysis of dendrite arm spacing (DAS), the diffusion distance of major elements between interdendritic/intercellular regions, and the core of the dendrite throughout the solidification of the weld metal. Tabaie et al. [14] reported on measuring the cooling rate ($\dot{\varepsilon }$), based on the primary DAS near the weld interface, using empirical Equation (4), which was also reported by Zhang et al. [19]:

(4) $${DAS}_{\left(\mu m\right)}=518.39{\left(\dot{\varepsilon }\right)}^{-0.592}.$$

For this specific purpose, it was assumed that the model constant values for IN686 were equivalent to those of IN718, given their similar thermal properties [20]. The DAS was quantified in the lower section of the weld metal (i.e., close to the weld interface and after the planar solidification morphology in Figure 6 and Figure 7).

**Figure 8:**
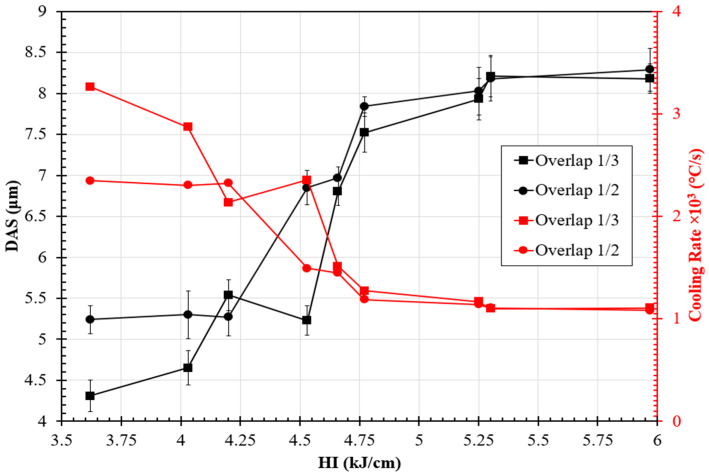
Variation in the DAS and corresponding calculated cooling rates in the bottom region (B) of the IN686 weld metal as a function of the HI and the weld overlap.

The DAS measurements and corresponding calculated cooling rate values were plotted, as shown in Figure 8, demonstrating the positive correlation between the HI and DAS and the negative correlation between the HI and cooling rate; in other words, the DAS increased with increases in the HI for both overlap conditions, resulting in slower cooling than expected. Moreover, the OT weld samples exhibited a smaller DAS than the OH samples, particularly in the lower heat inputs (3.62 and 4.03 kJ·cm^−1^), which indicates that the smaller overlap caused higher cooling rates in the weld metal due to a greater heat transfer than the steel substrate instead of the relatively hot adjacent weld bead. The cooling rates for the minimum weld heat input samples, OH3.62 and OT3.62, were calculated to be 2.35 × 10^3^ and 3.26 × 10^3^ °C·s^−1^, respectively, while the cooling rates for the maximum heat input samples, OH5.97 and OT5.97, were calculated to be 1.05 × 10^3^ and 1.12 × 10^3^ °C·s^−1^, respectively.

#### 3.2.3. The Weld Metal—Secondary Phase Formation

**Figure 9:**
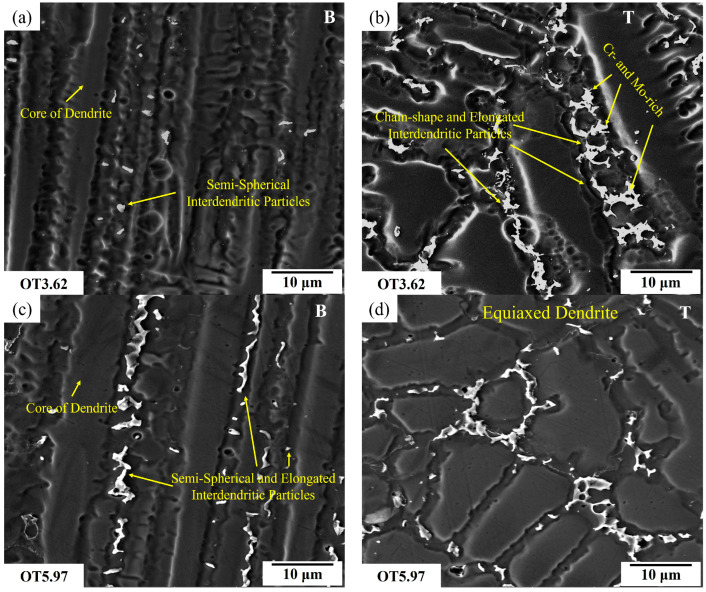
SEM micrographs of the (**a**,**c**) bottom (B) and (**b**,**d**) top (T) areas of OT weld overlay samples fabricated with heat inputs of (**a**,**b**) 3.62 kJ·cm^−1^ and (**c**,**d**) 5.97 kJ·cm^−1^.

The effects of the dilution and cooling rates on the formation of intermetallic particles in the weld metal were investigated by analyzing the microstructure within the weld metal from the weld interface (B) toward the top surface (T). Figure 9 shows the SEM micrographs and corresponding analyses of both the B and T regions of the weld metals of OT3.62 and OT5.97. The morphology of the interdendritic particles was found to change from the semi-spherical and discrete particles at the B region of OT3.62 (Figure 9a) to the elongated and chain-shaped particles at the T region of OT3.62, while both the T and B regions of OT5.97 exhibited an elongated particle morphology (Figure 9b–d). 

The quantitative particle analysis results from the bottom and top regions of the overlay for three different heat inputs and both overlaps are presented in Table 4. The results revealed that the size and volume fraction (V_f_) of these interdendritic particles increased from the B region to the T region. The microstructure of the B region was affected by a higher cooling rate and an enrichment of Fe. While the T region was not affected by Fe dilution, it was subjected to a lower thermal gradient, which could increase the volume fraction and the size of particles, as has also been reported in welded Inconel 718 superalloys [20].

The microsegregation of alloying elements in the IN686 superalloy was notably impacted by the heat input due the effect of the heat input on the cooling rate, DAS, and Fe dilution. As shown in Table 4 and Figure 9, the size and the V_f_ of the interdendritic particles increased with increases in the weld heat input. This indicates that increasing the heat input from 3.62 to 5.97 kJ·cm^−1^ (i.e., increasing the dilution and reducing cooling rate and thermal gradient) increases the segregation of alloying elements (i.e., heavy elements such as Mo and ~Cr) from the dendrite core to the interdendritic regions. 

Furthermore, Table 4 reveals that the size and the V_f_ of the intermetallic particles were also increased by reducing the weld overlap (OH to OT) at the same HI. For example, for the HI 3.62 kJ·cm^−1^, the V_f_ and the size of particles increased from 0.82% ± 0.13 and 0.65 ± 0.2 μm (OH) to 1.08% ± 0.14 and 2.94 ± 0.11 μm (OT) due to increased dilution in the OT sample. It should be noted that the cooling rate at OT3.62 was higher than OH3.62, which must have reduced the microsegregation, but the higher dilution in the OT samples had a dominant effect and increased the microsegregation.

**Figure 10:**
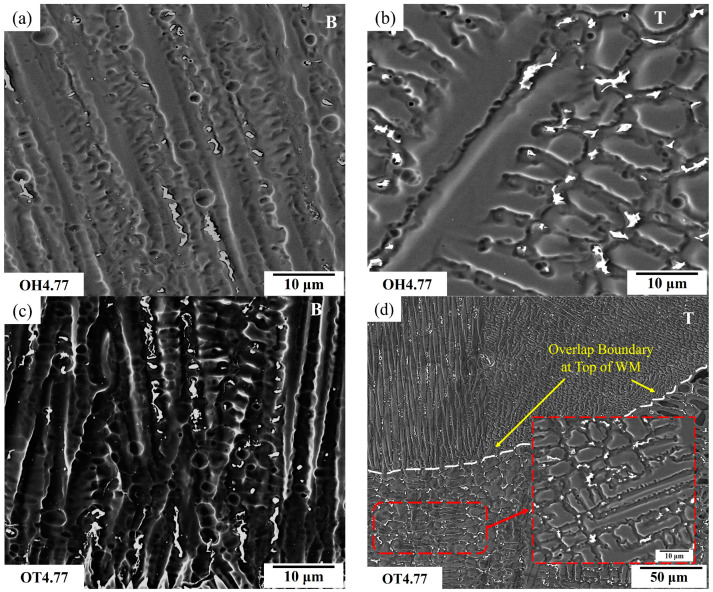
SEM micrographs of the (**a**,**c**) bottom (B) and (**b**,**d**) top (T) areas of the weld overlay samples for the (**a**,**b**) OH and (**c**,**d**) OT samples fabricated with a constant heat input of 4.77 kJ·cm^−1^.

Figure 10 compares the effects of weld overlap on the B and T regions of the weld metal microstructures when fabricated with a constant heat input of 4.77 kJ·cm^−1^. As presented in Table 4, reducing the overlap from OH to OT resulted in an increase of 25% and 278% in the V_f_ of the interdendritic particles in the B and T regions of the weld metal, respectively (which occurred due to increased Fe content/dilution). In addition, the size of these particles in the OT sample also increased in the B (48%) and the T (61%) regions compared to the OH sample, indicating the greater effect of weld overlapping (or less re-heating in OT samples) that occurred on the T region compared to the B region (which was dominated by the effect of the dilution level) of the weld overlay. In the overlap area, as the molten metal solidified, the heavy elements (e.g., Cr, Mo, and W) had a higher affinity for the liquid phase, while others tend to segregate toward the solid phase. The degree of overlap and cooling rates in the overlap boundary and the top layer can influence the extent of microsegregation [21]. Welding with larger overlaps causes reheating and a partial remelting of the former bead, resulting in a redistribution of segregated elements that can decrease the size and the fraction of interdendritic particles.

Table 5 presents the calculated partition coefficient ($k=\frac{C_{s}}{C_{L}}$) for the main alloying elements based on the EDS measurements in the bottom areas of samples. The composition of the solid phase at the commencement of solidification was denoted as Cs (which were evaluated by performing EDS point analysis on the dendrite core/centerline), and C_L_ = C_0_ represents the chemical composition of the liquid phase, which is typically equal to the nominal composition of the weld metal. The k values determined for Ni, Fe, and W were found to be greater than 1, indicating that these elements have a propensity to be assimilated into the solid phase during the solidification of the weld metal. Conversely, the k values calculated for Mo and Cr were found to be less than 1, thus signifying their tendency to segregate toward the liquid metal and interdendritic regions during solidification. Due to increasing the weld heat input and applying less overlap (1/3), the dilution was increased and the k values for Mo decreased, which is in agreement with prior reports [11]. Higher cooling rates in the OT samples (compared to the OH samples and at the same weld heat input) did not reduce the microsegregation in the OT samples, which indicates the stronger effect of dilution level than cooling rate on microsegregation. A slight decrease in the coefficient k for Cr and W was observed due to increasing the dilution and reducing the weld overlap. Maltin et al. [22] reported that the coefficient k for W decreased as the dilution of weld metal (IN686 superalloy) increased.

The decrease in the solubility of Mo and Cr in the Ni matrix, which is linked to the increasing Fe content within the solid solution resulting from dilution, may adversely affect the mechanical properties and corrosion resistance of the overlay layer [23]. Segregation of these elements promotes the formation of brittle intermetallic and TCP phases, which are deleterious to alloy performance [24].

**Figure 11:**
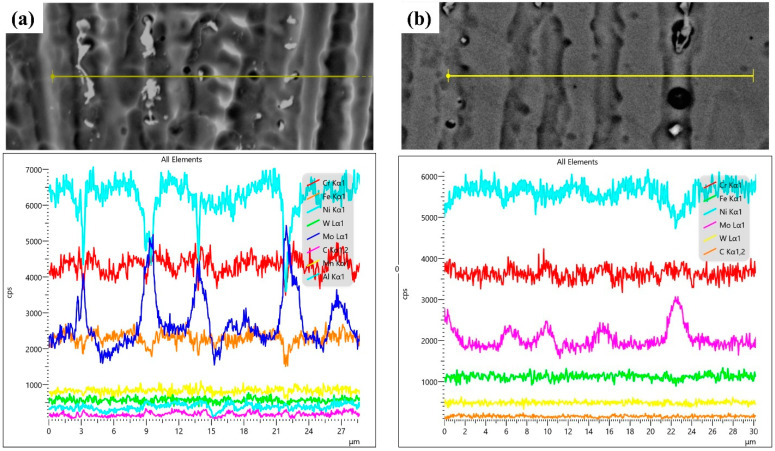
EDS line-scan analysis of the elemental variation in the dendrite core, interdendritic regions, and interdendritic particles in the bottom part of the weld metal for (**a**) OT4.77 and (**b**) OH4.77.

Figure 11 shows the SEM-EDS line-scan analysis depicting the variation of constituent elements in both the dendrite core and interdendritic regions within the bottom section of the weld metal for the OT4.77 (Figure 11a) and OH4.77 (Figure 11b) samples. It should be noted that the Mo was clearly enriched in the interdendritic regions, and the Ni was clearly depleted at the interdendritic region and particles. Other elements seemed to show similar behavior, but the signals were much lower. Table 6 presents the average chemical composition of the interdendritic precipitates analyzed by EDS (by controlling the time of spot analyzing). The obtained results were similar to the findings reported by Silva et al. [9], who utilized both TEM/EDS and SEM/EDS to analyze the same particles. For the OH samples, the Cr concentration in the interdendritic particles was slightly higher than its concentration in the IN686 filler wire (20.5%, Table 1). However, the Cr concentration in the interdendritic particles in samples OT4.77 and OT5.97, which had the highest d_c_ at ~14% (Fe dilution), was lower than its concentration in the filler wire. With an increase in both the heat input and dilution (more than ~14%), there was a distribution of microsegregated Cr across a higher fraction of particles, thus leading to a reduction in the proportion of Cr-rich particles. However, based on the average chemical composition of particles and compared with the literature [9,11,24,25], it appears that these Cr-rich (probably σ-phase) particles underwent conversion into other intermetallic particles (P or μ phase). This phenomenon aligns with the findings reported by Silva et al. [9] in the context of GTAW overlays on the IN686 superalloy, where a dilution level of 10–19% was measured.

Mina et al. [11] reported a reduction in the solubility of the Cr and Mo in the Ni-matrix of the GTAW IN686 superalloy, where the increasing Fe dilution was induced by higher weld heat inputs. The current investigation further revealed that a decrease in the tracks overlapping (OT samples) resulted in an increase in the interdendritic particles due to the microsegregation of Cr, Mo, and W, which was attributed to the elevated Fe dilution.

### 3.3. Thermo-Calc Simulation

**Figure 12:**
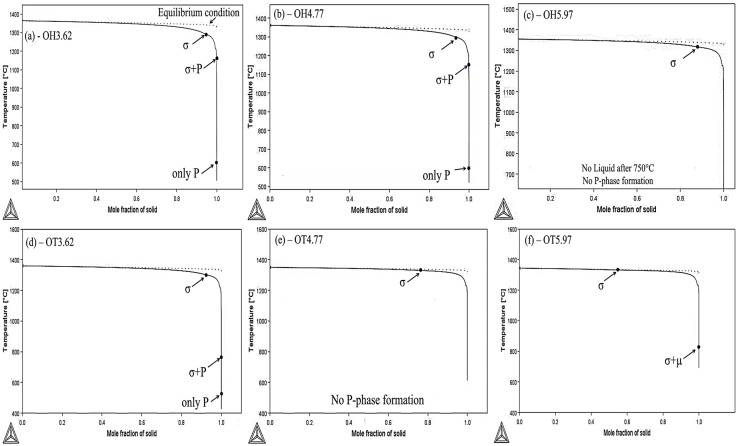
Predicted phase evolution (the mole fraction of a solid) vs. temperature (°C) diagram, which was calculated for non-equilibrium conditions based on the diluted IN686 weld metal composition for samples (**a**) OH3.62, (**b**) OH4.77, (**c**) OH5.97, (**d**) OT3.62, (**e**) OT4.77, and (**f**) OT5.97.

The solidification morphology of the weld metal (Figure 7, Figure 9 and Figure 10) and cooling rates (Figure 8) determined that the weld metal was solidified in non-equilibrium conditions (i.e., cellular and dendritic structures with only primary arm and cooling rates of up to ~3000 °C·s^−1^ at the bottom part of the weld metal). Therefore, Thermo-Calc simulations were performed using the Scheil equation and non-equilibrium conditions (Figure 12) as a complementary method and for comparative studies of the selected samples. The results of the solidification simulation are shown in Figure 12, with a particular emphasis on the evolution of topologically close packed (TCP) phases near the end of solidification. The predictions indicated that the formation of the P-phase was not predicted in samples welded with the highest levels of dilution (i.e., OH5.97, OT4.77, and OT5.97) under non-equilibrium cooling conditions. However, other TCP phases (the σ- and μ-phase) were predicted to form in these conditions due to the higher levels of heat input and Fe dilution. 

As shown in Figure 12e, the dilution level of OT4.77 (14.33%) was close to OT5.97 (14.29%), as reported in Table 3; as a result, the P-phase formation was not predicted for OT4.77, which can be attributed to the dc measurement that is based on measured Fe content, but the Cr, Ni, Mo, and W can vary from point to point in the EDS analysis in the weld metal near the weld interface.

While the OH5.97 sample had a slightly lower cooling rate (1.05 × 10^3^ °C·s^−1^) (Figure 9), the higher Fe dilution in OT5.97 (d_c_: 14.29%) compared to OH5.97 (d_c_: 11.21%) resulted in a μ-phase formation in the Thermo-Calc simulation. Therefore, the combination of a low cooling rate and the increased Fe dilution resulting from reduced weld overlapping can enhance the likelihood of TCP phase formation.

According to the average chemical composition of these particles reported in Table 6, and with the reactions predicted at the end of solidification in the Thermo-Calc simulations, the identity of the interdendritic particles can be predicted, as shown in the last column of Table 6, which agrees with the results presented in literature [9,11]. However, more precise characterization methods such as TEM were required to fully characterize and identify these phases. For example, the amount of Cr in the finer particles within the interdendritic regions, which are likely the P or μ phases, has been reported to be between 11.2% [9] and 17.8% [26,27]. The Mo had a relatively higher concentration in the interdendritic particles, ranging from 23.67% (OH3.62) to 41.46% (OT5.97). The microsegregation of the Mo content affected the microstructure by the formation of TCP precipitates (with the formula of (Cr, Mo)x (Ni, Co)y [24]) in two ways. First, increasing the Mo content increased the extent of the precipitation of the TCP phases (thus showing a correlation between Table 5 and Table 6); second, as the Mo content increased, the morphology of the TCP phase changed from solely plate-like or semi-spherical precipitates to a more chain-shaped (and blocky) morphology (Figure 10 and Figure 12).

The γ-phase (Ni-matrix) began to solidify and rejected some elements—particularly Mo, Cr, and W—in the liquid metal, primarily those within the interdendritic regions. These conditions favored the formation of the TCP phases, specifically the P and σ phases. The existence of precipitates similar to the σ-phase in the microstructure can be ascribed to at least two potential explanations. The initial explanation is that the solid-state transformation from the σ-phase to the P-phase takes place at a relatively rapid rate and cannot reach full equilibrium conditions (such as low heat input samples), resulting in an incomplete transformation and the persistence of the residual σ-phase. Another potential explanation pertains to minor variations found within the chemical composition of the weld metal volume, which stemmed from an incomplete mixing of the molten metal. This incomplete mixing can lead to the localized enrichment of elements like Fe and Cr, which play a pivotal role in the formation of the σ-phase. Consequently, in these specific regions, solidification of the remaining liquid into the σ-phase is probable, and this state persists during the cooling process, thus preventing a transformation into the P-phase. At the last stages of solidification and the solid-state transformation, it can be said that, due to the high temperature, there could be a solid state σ to P transformation followed by a subsequent P to µ-phase partial transformation. Mina et al. [11,28] employed Thermo-Calc analysis and revealed that a secondary reaction on the σ-phase occurred, and the P-phase was precipitated, due to a liquid to solid state transformation during the last stages of solidification.

Cieslak et al. [29] investigated the welding of alloys similar to the IN686 superalloy and observed that the chemical composition of coarser and chain-shaped particles, which was determined to be the σ-phase, contained higher concentrations of Cr and Fe but lower levels of Ni and Mo when compared to finer particles, which were determined to be the P and μ phases. These findings align with the observations made for the σ-phase examined in the microstructure of the GTAW overlaid IN686 alloy [9]. Silva et al. [9] reported that the electron valence/atom ratio (e/a) of the P-phase (7.39) is within the range favorable for μ-phase stabilization, which increases the formation tendency of the μ-phase near the P-phase concentration regions. However, the crystal structure and stoichiometry of the μ-phase changes, due to rejecting the Cr and Ni from the P-phase, resulted in an enrichment of Mo and W. Eventually, the μ-phase evolved as a result of the partial transformation of P to μ. Calculations conducted by Pan et al. [26] pointed to an almost complete conversion of the P-phase into μ-phase. However, the short exposure time of the weld metal at high temperatures during welding thermal cycles did not facilitate significant changes in the P-phase into the μ-phase. Consequently, this can result in the prevalence of the P-phase as opposed to the μ-phase within the microstructure of alloy IN686.

**Figure 13:**
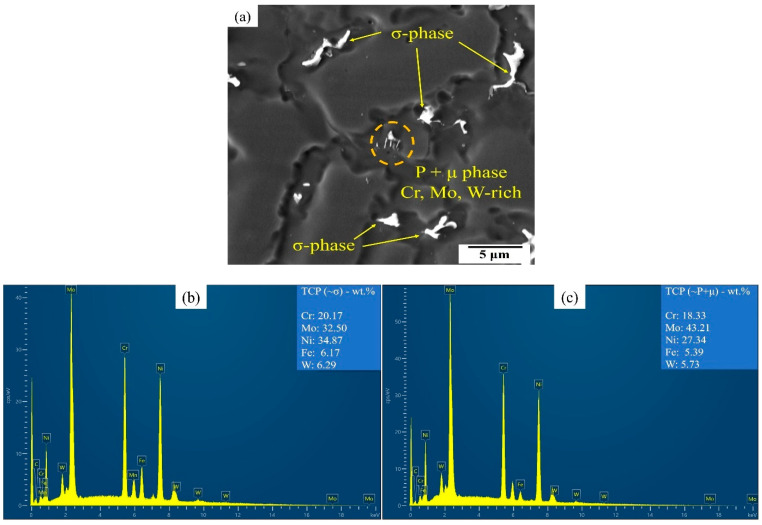
(**a**) SEM micrograph and (**b**,**c**) the EDS spectrums of several likely TCP phase IDs, which are labeled in the top part of the weld metal sample OT5.97.

Figure 13 shows a higher magnification SEM micrograph of the particles observed in the interdendritic region in the top region of the weld metal in the OT5.97 sample. The chemical composition of the finer particles, which had different shapes than the others, was analyzed by EDS spot analysis to be as follows: Cr: 18.33%, Mo: 43.21%, Ni: 27.34%, Fe: 5.39%, and W: 5.73% (Wt.%). These values were found to be similar to the P- and μ-phase compositions reported in other studies [9,11,24,30]. The particle’s shape was also the same as the P + μ phases detected by TEM/EDS analysis and reported by [9].

### 3.4. Hardness Measurement

**Figure 14:**
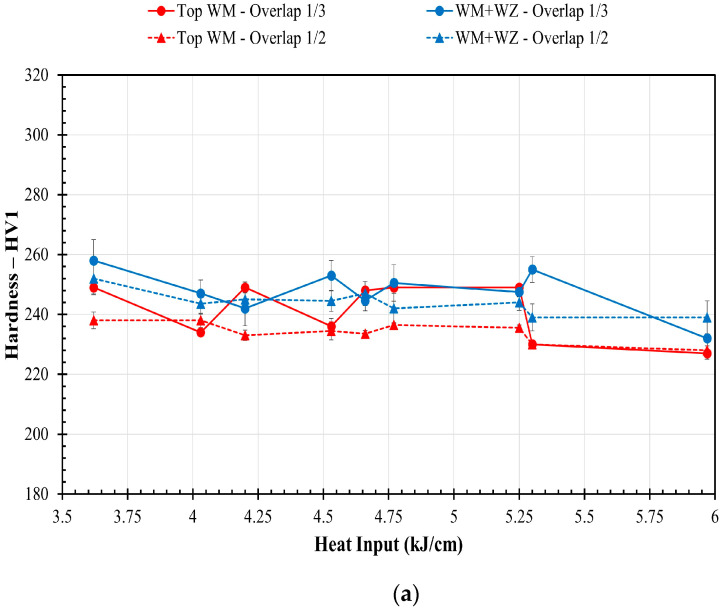

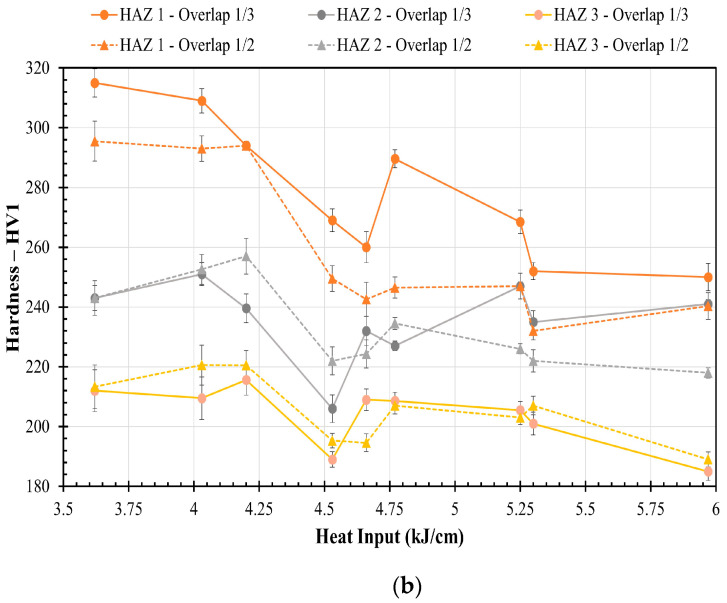
Vickers hardness (HV1) variation as a function of the welding heat input and track overlap amount in (**a**) the weld metal and (**b**) the HAZs.

Figure 14a shows the average hardness variation as a function of the weld heat input in the weld metal’s bottom and top regions for the two welding overlap conditions. The hardness generally decreased with increasing the heat input in both the weld metal zones for both overlaps. In general, the hardness of the bottom region (WZ + WM) was higher than the top region in all the samples and both weld overlaps. The OT samples generally exhibited slightly higher hardness (~3% at the WZ + WM and ~6% for the top part) compared to the OH samples with the same heat input, which was attributed to a shorter DAS in the OT samples compared to the OH samples.

Figure 14b shows the hardness variation in the various HAZs (Figure 5) against the HI for two welding overlaps; a detailed description of the various HAZs was provided in [14]. The hardness measurement of the unaffected base metal was consistent across all samples, measuring at 169.0 ± 3.1 HV. The hardness levels exhibited a general decline as the weld heat input increased, which was observed in both weld overlaps across all HAZs. In HAZ 1, which corresponded to the coarse-grained HAZ directly adjacent to the fusion zone, the hardness levels decreased when increasing the weld overlap from 1/3 (OT) to 1/2 (OH) due to the greater heat input that was injected into the steel welded by a larger overlap (OH samples). However, in HAZ 2 and HAZ 3, which corresponded to the fine-grained HAZs that were further removed from the fusion zone, the hardness trends depicted that the hardness was slightly higher for OH vs. OT. For samples welded by the heat input from 3.62 to 4.65 kJ·cm^−1^, the hardness measurements in HAZ 1 were found to be higher than the weld overlay. This can be attributed to the higher cooling rates that result from the lower heat input, which, in turn, lead to the presence of a greater amount of upper bainite and lath martensite, as reported in [14]. Furthermore, the hardness measurements indicated that, in the welded samples with higher heat inputs that resulted in increased dilution, the hardness of HAZ 1 was reduced. This decrease can be attributed to the Fe dilution in the weld metal, grain coarsening, and slower cooling rates. The increased dilution of Fe in the weld metal at higher heat inputs could also contribute to the decreased hardness in the overlay, via the formation of brittle TCP phases, and a diminished solid solution strengthening of the alloy, as was discussed in Section 3.3 and evaluated by previous investigations [30].

## 4. Conclusions

The objective of the current investigation was to study the effect of changing the weld process parameters and weld overlap distances on the microstructure and properties of the IN686 GMAW overlays. The weldment quality, microstructure, and hardness were affected by the weld overlay parameters. Various evolutions of microstructural features of the GMAW IN686 superalloy on this low-carbon steel, specifically the precipitation of TCP phases, were investigated based on the obtained and discussed results, the main findings of which can be summarized as follows:

- Although reducing the weld overlap from 1/2 (OH) to 1/3 (OT) can decrease the required number of weld tracks in the overlay on a substrate (e.g., from 12 to 10 in the current study), less overlapping increases the level of dilution and intermetallic phase formation. Therefore, weld overlapping at 1/2 (i.e., 4–5 kJ·cm^−1^) results in better weld overlay properties than overlapping at 1/3.
- The microstructure evolution is strongly influenced by the weld parameters and overlap through the corresponding level of dilution and cooling rate. In general, greater levels of dilution led to a greater volume fraction and larger size of intermetallic/interdendritic phases.
- Increasing the heat input increases the Fe dilution from the base metal into the weld metal, as well as reduces the cooling rate and consequently increases the DAS. This leads to greater Cr and Mo microsegregation in the interdendritic regions and the formation of a higher fraction of intermetallic phases. Although shorter weld overlaps (1/3) increased the cooling rates (at the weld interface), it also induced higher Fe dilution, which consequently doubled the TCP phases volume fraction in the IN686 overlays.
- The specific phase evolution at the end of solidification depends on both the heat input and the track overlap. Variations in the volume fraction, particle size, and chemical composition were measured for different weld overlay conditions. The Thermo-Calc predictions indicated that these particles are likely the P-, σ-, and µ- type TCP phases.
- As a result of the elevated dilution of the weld metal (i.e., increased Fe content due to the diffusion and mixing in the molten weld pool), the hardness of the overlay was less than the HAZ, and HAZ 1 was significantly harder than the base metal due to high cooling rates. Lastly, increasing the heat input reduced the HAZ hardness but increased overall HAZ thickness.

## Figures and Tables

**Table 1:** The nominal chemical compositions of the as-received filler metal and substrate metal (wt.%).

Materials	Ni	Fe	Al	Mn	C	Cr	Mo	Ti	W	Si	Cu	S	P
**Inconel 686 (Filler wire)**	Bal.	0.28	0.27	0.27	0.007	20.50	16.4	0.09	3.60	0.05	0.01	<0.001	0.001
**SA516-Gr.70 (Substrate)**	0.160	Bal.	-	1.184	0.157	0.170	-	0.002	-	0.343	0.131	0.002	0.012

**Table 2:** The DoE and calculated HI for the two weld overlap conditions (OH and OT).

Sample	V (V)	TS (cm/min)	HI (kJ/cm)
OH4.53 & OT4.53	19	40.6	4.53
OH4.03 & OT4.03	19	45.7	4.03
OH3.62 & OT3.62	19	50.8	3.62
OH5.25 & OT5.25	22	40.6	5.25
OH4.66 & OT4.66	22	45.7	4.66
OH4.20 & OT4.20	22	50.8	4.20
OH5.97 & OT5.97	25	40.6	5.97
OH5.30 & OT5.30	25	45.7	5.30
OH4.77 & OT4.77	25	50.8	4.77

**Table 3:** The weld geometries and the geometric and compositional dilutions of the selected welded samples (BW: bead width, P: penetration, and R: weld reinforcement).

S#	HI (kJ/cm)	BW (mm)	P (mm)	R (mm)	HAZ (mm)	d_g_ (%)	d_c_ (%)
**Overlap 1/2**							
**OH3.62**	3.62	12.5 ± 0.20	0.25	2.50	1.82	6.01	5.26
**OH4.77**	4.77	13.1 ± 0.26	0.48	2.95	2.30	8.34	7.76
**OH5.97**	5.97	12.7 ± 0.17	0.63	3.40	2.90	13.63	11.21
**Overlap 1/3**							
**OT3.62**	3.62	11.9 ± 0.18	0.36	2.70	1.82	8.38	7.30
**OT4.77**	4.77	12.1 ± 0.23	0.60	2.40	2.20	15.39	14.33
**OT5.97**	5.97	12.0 ± 0.18	0.60	2.85	2.80	14.03	14.29

**Table 4:** Variation in the volume fraction and size of interdendritic particles at the bottom and the top regions of the weld metals fabricated with different heat inputs and track overlap values.

S#	Bottom of Weld Metal		Top of Weld Metal	
	V_f_ (%)	Size (μm)	V_f_ (%)	Size (μm)
**Overlap 1/2**				
**OH3.62**	0.82 ± 0.13	0.65 ± 0.20	1.29 ± 0.08	1.22 ± 0.26
**OH4.77**	1.37 ± 0.05	1.02 ± 0.26	1.49 ± 0.06	1.49 ± 0.06
**OH5.97**	1.65 ± 0.21	1.34 ± 0.43	1.92 ± 0.18	1.92 ± 0.18
**Overlap 1/3**				
**OT3.62**	1.08 ± 0.14	1.30 ± 0.80	3.86 ± 0.11	2.20 ± 0.40
**OT4.77**	1.62 ± 0.17	1.50 ± 0.30	4.27 ± 0.16	2.10 ± 0.30
**OT5.97**	2.94 ± 0.11	4.30 ± 1.10	5.02 ± 0.09	5.90 ± 1.40

**Table 5:** Calculated partition coefficient (k) of the main alloying elements in the bottom areas of the weld overlay samples welded with different heat inputs and overlapping.

S#	d_g_ (%)	d_c_ (%)	*k*				
			Cr	Fe	Mo	Ni	W
**Overlap 1/2**							
**OH3.62**	6.01	5.26	0.974	1.041	0.832	1.060	1.071
**OH4.77**	8.34	7.76	0.952	0.932	0.841	1.102	1.065
**OH5.97**	13.63	11.21	0.948	1.046	0.799	1.070	1.015
**Overlap 1/3**							
**OT3.62**	8.38	7.30	0.971	1.052	0.811	1.063	1.055
**OT4.77**	15.39	14.33	0.931	1.032	0.749	1.022	1.001
**OT5.97**	14.03	14.29	0.928	1.029	0.785	1.014	1.007

**Table 6:** Chemical composition of the interdendritic particles observed in the bottom part of the weld metal, as measured by EDS analysis (wt.%).

S#	Cr	Fe	Mo	Ni	W	Proposed Phase (s)
**Overlap 1/2**						
**OH3.62**	20.29 ± 1.01	5.17 ± 0.67	23.67 ± 1.64	46.99 ± 1.83	3.88 ± 0.66	σ + ~P
**OH4.77**	22.03 ± 0.60	5.81 ± 0.72	28.40 ± 1.25	37.92 ± 2.54	5.68 ± 0.78	σ
**OH5.97**	23.95 ± 0.43	5.78 ± 0.91	31.70 ± 1.62	32.78 ± 1.79	5.79 ± 0.23	σ
**Overlap 1/3**						
**OT3.62**	21.58 ± 0.74	5.12 ± 0.81	34.67 ± 1.11	33.62 ± 2.14	5.01 ± 0.45	σ + ~P
**OT4.77**	19.80 ± 1.48	6.27 ± 0.48	39.34 ± 1.46	28.14 ± 1.78	6.45 ± 0.77	σ + ~P
**OT5.97**	16.42 ± 1.89	6.35 ± 0.23	41.46 ± 1.12	28.96 ± 1.38	6.81 ± 0.68	σ + ~μ + ~P

## Data Availability

The original contributions presented in the study are included in the article, further inquiries can be directed to the corresponding author.
